# A Randomized, Triple-Blind, Placebo-Controlled, Add-On Clinical Trial to Evaluate the Efficacy of *Emblica officinalis* in Uncontrolled Hypertension

**DOI:** 10.1155/2020/8592869

**Published:** 2020-10-07

**Authors:** Samad Ghaffari, Maryam Navabzadeh, Mojtaba Ziaee, Ali Ghobadi, Roshanak Ghods, Fataneh Hashem-Dabaghian

**Affiliations:** ^1^Tabriz University of Medical Sciences, Tabriz, Iran; ^2^Research Institute for Islamic and Complementary Medicine, School of Persian Medicine, Iran University of Medical Sciences, Tehran, Iran; ^3^Medicinal Plants Research Center, Maragheh University of Medical Sciences, Maragheh, Iran

## Abstract

*Introduction. Emblica officinalis* (EO) has some cardiovascular effects, and there are some animal studies that show its antihypertensive effect. This study was conducted to determine the effect of combination of EO with standard therapy on the systolic blood pressure (SBP) and diastolic blood pressure (DBP) in patients with uncontrolled hypertension. *Materials and Methods.* This was a randomized, triple-blind, placebo-controlled, 8-week study. Ninety-two patients with uncontrolled hypertension despite taking hypotensive drugs were randomly assigned into two groups to take EO (500 mg/TDS after meal) or placebo in combination with standard antihypertensive drugs. After 2, 4, 6, and 8 weeks of intervention, SBP and DBP and heart rate (HR) were measured. Data were analyzed by SPSS software using repeated measures ANOVA. *Results.* Eighty-one patients (41 in the drug group and 40 in the placebo group) completed the study for 8 weeks and were analyzed. The mean ± standard deviation of age was 53.64 ± 10.01 years. SBP decreased as 15.6 ± 8.23% in the EO group and 6.3 ± 7.49% in the placebo group (*P* < 0.001). DBP decreased as 12.3 ± 7.87% and 3.88 ± 7.98%, respectively (*P* < 0.001). Time effect was not significant, but the group effect was significant (*F* = 13.875, *P*=0.001 for systolic BP and *F* = 18.948, *P* < 0.001 for diastolic BP). No side effects were reported during the study. *Conclusion.* Eight-week combination therapy of EO with standard antihypertensive drugs significantly reduced the SBP and DBP more than placebo in patients with uncontrolled hypertension.

## 1. Introduction

Hypertension is one of the important public health and economic problems “worldwide”. The prevalence of hypertension is high both in developing and developed countries [1, 2]. It has been estimated that 60% of adults will have hypertension by the year 2025 [3]. Based on the results of a systematic review until 2012, the overall prevalence of hypertension in Iran was about 22% [4]. Another systematic review in 2017 reported the prevalence of hypertension in Iran as about 17% [5].

Hypertension is an important predictor of premature death and disability and plays a key role in the mortality and morbidity from cardiovascular diseases and cerebrovascular accidents [6].

In 2010, high blood pressure was one of the 5 or 6 leading risk factors for Global Burden of Disease (GBD) worldwide, as assessed by DALYs [7].

The goal of the World Health Organization (WHO) is to reduce the prevalence of hypertension to 25% [8]. Hypertension is the most prevalent risk factor for cardiovascular diseases and death globally [9]. Despite the availability of various types of antihypertensive drugs, control of hypertension is reported in 6 to 30% of the population in different societies [3]. Hypertensive patients tend to use complementary medicine with various reasons [10].

Several plants are used in herbal medicine to lower blood pressure, and some plants have been studied for this purpose.

*Emblica officinalis Gaertn* (EO) or amla (family Euphorbiaceae) is a medium-sized tree, native to India, and is cultivated in Pakistan, Uzbekistan, Sri Lanka, Southeast Asia, China, and Malaysia. Its other names are *Phyllanthus emblica* Linn, *Emblica pectinata* Ridl, emblic myrobalan, and Indian gooseberry [11]. The dried fruit is a common imported herbal product in the herbal markets of Iran. The fresh fruits resemble green sour plums about the size of a walnut.

The EO fruit is the most commonly used part of the plant for treatment of various diseases in the Ayurveda, Unani, and Persian medicines (PM) [11]. In the perspective of PM, amla is said to be a cardiotonic because it has astringent properties and can strengthen the cardiac tissue [12].

In a systematic review of Hashem-Dabaghian et al. in 2018, the cardiovascular effects of this plant had been investigated [13]. According to the results of this review, EO has antiatherogenic, antihypertensive, anti-inflammatory, antioxidant, antiplatelet, vasodilator, and lipid deposition inhibitory effects. Moreover, it improves vascular endothelial function.

Bhatia et al. reported the effect of EO on DOCA-salt-induced hypertension [14]. In the study of Patel and Goyal, the effect of EO was evaluated in the rat model of diabetic-induced myocardial dysfunction, and its effect on high blood pressure was observed as a secondary outcome [15].

In the quasi-experimental study of Gopa et al., the effect of EO on hyperlipidemic patients had been investigated, and its effect on hypertension was observed [16].

In the thesis of Srivastava, EO powder was compared with vitamin-C. A significant reduction in the systolic and diastolic BP was reported in the EO group compared with other groups [17].

There has been no proper evidence to make a decision about the efficacy and safety of EO in hypertensive patients; therefore, this study was designed to compare the effect of EO fruit with placebo in patients with hypertension.

## 2. Materials and Methods

### 2.1. Preparation of the Drug and Placebo

EO fruit was purchased from a local herbal market in Tehran (Iran). It was authenticated by a botanist, and a voucher specimen (voucher number: PMP-1611) was stored at the Herbarium of the Faculty of Pharmacy, Tehran University of Medicinal Sciences, Tehran, Iran. The fruits were washed with tap water, dried under shade, and powdered in a metallic laboratory mill. The dried EO fruit powder was scaled and filled in capsules (500 mg). Placebo was made from the wheat starch powder and filled in capsules, and they were of the same color and size.

### 2.2. Participants

Patients were assessed for eligibility. The inclusion criteria were age range between 18 and 80 years, BMI < 30, SBP ≥ 140 mm Hg, DBP ≥ 90 mm Hg or both, and SBP ≥ 150 mm Hg for patients older than 60 years. Despite the use of antihypertensive drugs (maximum of 2 drugs for at least 8 weeks), a minimum of one month must have elapsed from the start of medication [18].

The exclusion criteria were new patients, SBP > 180, DBP > 110, secondary hypertension, history of hypertension complications, end-organ damage (EOD), pregnancy or breastfeeding, history of diabetes mellitus, cardiac, hepatic, or renal function impairment, allergy to the product under study, participation in any other clinical trial at the same time or within the last 30 days, drug abuse (alcohol consumption, etc.), coagulopathy, using warfarin, heparin, clopidogrel, and other antiplatelet agents, and NSAIDs.

The attrition criteria were any changes in the dosage of antihypertensive drugs, diet or exercise, using another drug during the study period, sudden increase in the blood pressure more than 15 mm Hg at any time of the study, blood pressure greater than or equal to 200/140 mm Hg, any side effects, and poor compliance.

The sample size was calculated employing the formula for comparing two means, considering type 1 error = 0.5, power = 80%, Cohen's *d* effect size = 0.65 (based on the results of our pilot study) for the SBP and DBP, and attrition rate = 20%. Forty-six patients were assigned to each group. [Cohen's *d* = (*M*_2_ − *M*_1_) ⁄SD_pooled_, SD_pooled_ = √ ((SD_1_^2^ + SD_2_^2^) ⁄2), *n* = (*Z* 1 − *α*/2 + *Z*1 −*β*)^2^.1/*d*^2^].

### 2.3. Study Design

This triple-blind, placebo-controlled, randomized clinical trial was performed at the cardiovascular clinic of Tabriz (Iran). The registration code in the Iranian Center for Clinical Trials is IRCT20090527001957N7.

After confirming the eligibility and filling the informed consent form, the patients were assigned into two groups through block randomization. Blocks of four (AABB, ABAB, BABA, BBAA, ABBA, and BAAB) were randomly selected to make the randomization list. Sequentially numbered opaque sealed envelopes were used for randomization concealment. The drug and the placebo were administered to the patients in similar packages and the same dose (containers were equal in weight and similar in appearance). There were letters A, B, C, or D on the containers, where A and C were EO capsules and B and D were placebo. The patients, the researcher, and data analyzer were blinded to the content of the containers.

### 2.4. Interventions

Both groups continued their previous antihypertensive treatments. Complementary intervention in group 1 was EO capsules (500 mg) three times a day after meal (13, 19) and in group 2 was the placebo capsules three times a day after meal for 8 weeks. Patients were reminded once a week through telephone to adhere to their medications.

### 2.5. Outcomes

Changes of SBP and DBP were the primary outcomes which were measured with calibrated mercurial sphygmomanometer (Riester, Model: 0124, Germany) using the appropriate cuff while observing the standards of BP measurement (10 minutes rest, half an hour after smoking or taking caffeine, sitting position, the nondominant arm was bare and on the heart level).

Measurements were repeated 3 times with intervals of 3 minutes, and the average systolic and diastolic pressure was recorded for each patient at each visit. The setting of BP measurement remained constant at baseline and at the end of the study.

Response to treatment was defined as SBP < 140 and/or DBP < 90 or a reduction of SBP by more than 10 mm Hg and/or DBP reduction of more than 5 mm Hg [18]. Other outcomes were heart rate (HR) and blood biochemical factors.

To measure the heart rate after 10 minutes of rest, the heart rate was measured for 30 seconds.

SBP and DBP were measured at baseline and at the 2^nd^, 4^th^, 6^th^, and 8^th^ week.

HR, fasting blood sugar (FBS), serum cholesterol and triglyceride (TG), alanine aminotransferase (ALT), aspartate aminotransferase (AST), BUN, and creatinine were measured at baseline and at the 8^th^ week.

To measure the medication adherence, patients were asked to mark the medications they have used in a checklist. Moreover, patients had to bring their remaining medications back at the next visit. Taking less than 20% of medications was considered as poor adherence [19].

### 2.6. Statistical Analysis

The data were analyzed by SPSS software (version 17). The mean ± standard deviation was utilized to describe the quantitative variables, and number and frequency percentage were employed for the qualitative variables. Comparison of qualitative variables between groups was done using the chi-square test. Quantitative variables were compared between groups using *t*-test.

To assess the effect of the time and treatment on the systolic and diastolic pressure, repeated measures ANOVA was used. Normal distribution of systolic and diastolic pressure was confirmed by Kolmogorov–Smirnov test. Mauchly's test was used to assess the sphericity. *P* values less than 0.05 were considered as statistically significant.

## 3. Results

**Figure 1:**
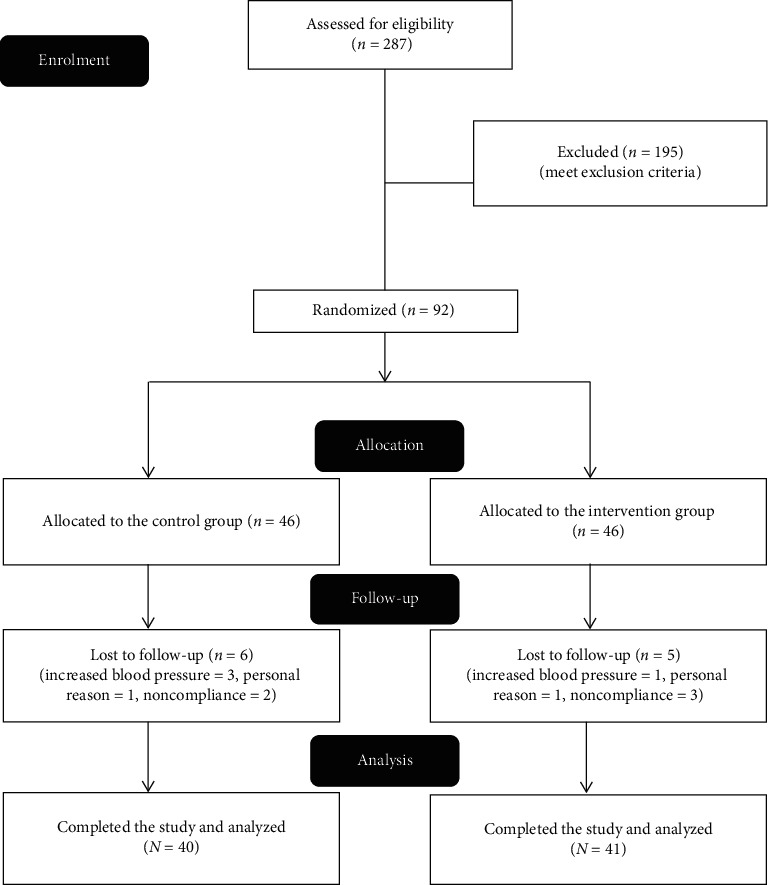
The trial flowchart.

Of the 287 patients who were assessed for eligibility, 92 patients were enrolled in the study and randomly assigned into two groups. Eighty-one patients (41 in the drug group and 40 in the placebo group) completed the study for 8 weeks and were analyzed. The CONSORT flow diagram and causes of the attrition are presented in Figure 1.

Age of participants ranged from 35 to 74 years.  Table 1 presents the baseline characteristics of the participants. Two groups were identical in the frequency of comorbidities such as diabetes and hyperlipidemia.

The number and median of antihypertensive drugs were 0–2 and 1 in both groups. The amounts of SBP and DBP and their changes are presented in Table 2.

Table 2 represents a further decrease of SBP and DBP in the EO group compared with the placebo group. Percentage of SBP decrease was 15.6 (8.2) mmHg in the EO group and 6.3 (7.4) mmHg in the placebo group (*P* < 0.001). Percentage of DBP decrease was 12.3 (7.87) and 3.88 (7.98) mmHg, respectively (*P* < 0.001). The results of the repeated measures ANOVA adjusted for the corresponding baseline BP and age are presented in Table 3.

Table 3 shows that the time effect was not significant, but the group effect was significant in reducing both SBP and DBP. EO add-on therapy significantly reduced the patients' SBP and DBP.

Table 4 presents the frequency of response to treatment (as defined in the method section).

Table 4 shows that the frequency of response to treatment was significantly higher in the EO group with the DBP reduction of more than 5 mm Hg. No side effects were reported during the study. The results of *t*-test for comparison between groups did not reveal any statistically significant difference between groups for HR, FBS, cholesterol and TG, ALT, AST, BUN, and creatinine at baseline and 8^th^ week.

## 4. Discussion

This is the first placebo-controlled clinical trial to examine the effect of EO add-on therapy on BP. The results of this study revealed that EO in combination with standard antihypertensive drugs reduced the systolic and diastolic blood pressure more than placebo in patients with uncontrolled hypertension. The frequency of response to treatment was significantly higher in the EO group. No significant complication was reported. EO has an astringent effect, but constipation was not observed if taken after meal. As in our study, nobody suffered from this disorder. In the case of constipation, sweet almond oil can be used [20].

There are some animal and a few low-quality studies that show the effect of EO on blood pressure [14–16, 21]. The main objective in the study of Usharani et al. was the effect of the *Phyllanthus emblica* (PE) extract on endothelial dysfunction and biomarkers of oxidative stress in patients with type 2 diabetes mellitus. Blood pressure changes were observed, and reduction of the endothelial reflection index showing the improvement of endothelial function was said to be attributed to the anti-inflammatory and antioxidant action of PE [21].

Gopa et al. evaluated the hypolipidemic efficacy of amla (*Emblica officinalis*), and reduction of blood pressure was also observed [16]. In addition to inclusion criteria, the small sample size and the lack of randomization were the limitations of these studies. The advantages of our study are the inclusion criteria, random allocation, blinding, and presence of the placebo group. The mechanism of the antihypertensive effect of EO was studied in some previous research studies. For instance, Fatima et al. evaluated the effect of the *Phyllanthus emblica* (PE) extract on cold pressor-induced cardiovascular changes in healthy people and observed the reduction of arterial stiffness and radial and aortic BP in the PE extract group [22].

EO is rich in phenols (gallic acids, methyl gallate, ellagic acid, and trigalloyl glucose) and flavonoids (quercetin and kaempferol) [13]. Previous studies suggest that taking polyphenol-rich foods can have good effects on cardiovascular diseases including hypertension and endothelial and platelet functions [23, 24]. Moreover, some animal studies demonstrated the effect of EO on the regression of aortic plaques and reduction of aortic atheromatous plaques [25–27].

Nowadays, scientists believe the effect of oxidative stress on blood pressure [28, 29], and EO can be effective in the prevention or treatment of hypertension through its strong antioxidative properties [30]. More importantly, EO is a plant with the ACE (angiotensin-converting enzyme) inhibitor and diuretic activity which can explain its antihypertensive effect [31, 32]. The strength of the present study was the triple-blind design and checking the medication adherence.

One of the limitations of our study was the lack of use of Holter monitoring of BP because of the high costs, and it is suggested for the future studies. Another limitation of the study was the lack of checking the adherence to antihypertensive drugs at the beginning of the study (we just asked patients about their adherence to their antihypertensive medications). In addition, we were not able to explain the effect of EO alone on hypertension because it was an “add-on” study.

Evaluation of the antihypertensive effect of EO in comparison with standard antihypertensive drugs can be mentioned in future trials. In addition, the antihypertensive effect of EO in higher stages of hypertension could be evaluated. If it is approved, EO could be applied in hypertensive patients as a safe and effective intervention.

## 5. Conclusion

Eight-week intervention with EO plus standard antihypertensive drugs significantly reduced the systolic and diastolic blood pressure more than placebo in patients with uncontrolled hypertension.

## Figures and Tables

**Table 1:** Baseline characteristics of participants.

	EO group (*n* = 41)	Control group (*n* = 40)	*P* value
Age (year)	56.05 ± 10.02	51.18 ± 9.5	0.02
(mean ± SD)			
Sex, *n* (%)	Male: 20 (48.8)	25 (62.5)	0.26
	Female: 21 (51.2)	15 (37.5)	
Marriage, *n* (%)	Single: 0	2 (5)	0.24
	married: 41 (100)	2 (5)	
BMI (mean ± SD)	28.08 (4.8)	27.04 (3.9)	0.3
Duration of HTN (months) (mean ± SD)	85.57 (83.36)	69.16 (65.25)	0.51
Duration of use of anti-HTN drugs (months) (mean ± SD)	65.47 (75.94)	60.85 (67.21)	0.84
Smoking, *n* (%)	7 (17.1)	11 (27.5)	0.29

EO: *Emblica officinalis*, SD: standard deviation, BMI: body mass index, and HTN: hypertension.

**Table 2:** Blood pressure and heart rate in each group during the study.

Time	Systolic blood pressure			Diastolic blood pressure		
	EO	Placebo	*P* ^*∗*^	EO	Placebo	*P* ^*∗*^
	Mean (±SD)	Mean (±SD)		Mean (±SD)	Mean (±SD)	
Baseline	152.7 (9.5)	146 (8.23)	0.001	93 (7.6)	91.4 (7)	0.31
Week 2	141.2 (15.3)	138.3 (13.8)	0.55	83.4 (8.5)	83.7 (9.6)	0.92
Week 4	133 (13.7)	139.1 (12.8)	0.17	82.1 (5.2)	85.8 (8.2)	0.11
Week 6	134.4 (15.8)	142.5 (11.5)	0.08	79.6 (8.2)	89.5 (8.8)	0.001
Week 8	126.7 (13.8)	136.7 (12.8)	0.009	81.2 (6.3)	87.6 (7.6)	<0.001

EO: *Emblica officinalis*; SD: standard deviation. ^*∗*^*t*-test.

**Table 3:** Time, treatment, time-treatment interaction, and baseline effect on systolic and diastolic blood pressure and age.

Effect	Systolic blood pressure		Diastolic blood pressure	
	*F*	*P*	*F*	*P*
Time	0.702	0.5	0.430	0.7
Treatment	13.875	0.001	18.948	<0.001
Time^*∗*^treatment interaction	1.709	0.3	16.428	<0.001
Baseline BP	16.809	<0.001	50.602	<0.001
Age	0.007	0.93	3.147	0.085

BP: blood pressure.

**Table 4:** Response rate in each group.

	Group	Response 1, *n* (%)	*p*	Response 2, *n* (%)	*p*
SBP	EO	34 (82.9)	0.048	35 (85.4)	0.001
	Placebo	25 (62.5)		20 (50)	
DBP	EO	39 (95.1)	0.007	34 (82.9)	<0.001
	Placebo	29 (72.5)		18 (45)	

EO: *Emblica officinalis*, Response1: SBP < 140 and/or DBP < 90, Response2: reduction of SBP by more than 10 mm Hg, SBP: systolic blood pressure, and DBP: diastolic blood pressure.

## Data Availability

The data used to support the findings of this study are available from the corresponding author upon request.
